# A case of non-invasive serous adenocarcinoma at unilateral fimbria with spread to the peritoneal/uterine cavity: case report

**DOI:** 10.1186/1746-1596-4-43

**Published:** 2009-12-05

**Authors:** Yuki Fukumura, Akiko Masaoka, Toshio Naito, Miki Kimura, Takashi Yao

**Affiliations:** 1 Department of Human Pathology, Juntendo University, Tokyo, Japan; 2 Department of Tumor and Pathology, Juntendo University, Tokyo, Japan; 3 Department of General Medicine, Juntendo University, Tokyo, Japan; 4 Department of Gynecology and Obstetrics, Juntendo University, Tokyo, Japan

## Abstract

Recently, fimbriae have been identified as a possible arising site for the pelvic serous carcinoma (PSC) both in BRCA-positive and BRCA-negative women. Although non-invasive (intraepithelial) serous adenocarcinoma of the fimbria has been found in specimens obtained from prophylactic salphingo-oophorectomies in BRCA-positive women, there has not been any case report in clinical situation, since this type of tumor is usually detected after stromal invasion/widespread dissemination. We describe a 67-year-old woman with non-invasive serous adenocarcinoma located solely in the left fimbria. This case may suggest the benefit of endometrial cytology and detailed gross examination of fimbria for the early detection of fimbrial carcinoma. This case may provide evidence suggesting fimbrial intraepithelial adenocarcinoma is one cause of PSC.

## Background

Pelvic serous carcinoma (PSC) has been presumed to arise in three different locations in the female pelvis [1]: the ovary (serous ovarian carcinoma), the endosalpinx (serous fallopian tube carcinoma) and on the peritoneal surface (primary peritoneal serous carcinoma). Among the three, primary cancer of the fallopian tube has previously been estimated to be very rare [2].

Recently, tubal fimbriae have been shown as the common site for early PSC (intraepithelial carcinoma) in women with BRCA mutations [3]. Also, the potential role of the fimbriae in primary PSC has been suggested [4,5], irrespective of BRCA-status. A few case of fimbrial adenocarcinoma, though invasive, has also been reported in surgical material resected for benign gynecological tumor at other sites [6].

We present a case of PSC caused by non-invasive serous adenocarcinoma involving the unilateral fimbria. To our knowledge, this is the first case report describing non-invasive, serous adenocarcinoma originating in the fimbria and spreading to the pelvic/intrauterine cavity in a clinical situation.

## Case presentation

**Figure 1:**
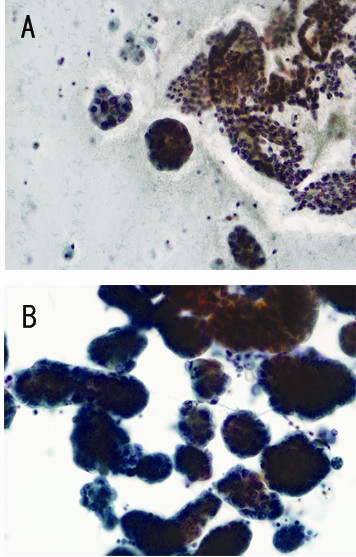
**Cytology of the tumor**. (A) Endometrial cytology shows nests of atypical cells among the sheets of well-organized, non-atypical endometrial cells. (B) Intraoperative cytology of the ascites shows abundant atypical cells with peripherally-located and pleomorphic nuclei in clear background. [Papanicalau stain, A; × 40, B; ×60]

A 67-year-old Japanese women, gravida 2, para 2, visited our hospital for regular (once 2 years) gynecologic screening. She had no family history for ovarian/breast carcinoma. There were no remarkable findings on physical examination/trans-vaginal ultrasonography. Endometrial cytology demonstrated abundant nests of adenocarcinomatous cells with peripherally located nuclei among sheets of non-atypical endometrial cells (Fig. 1A). Endometrial biopsy resulted in insufficient materials with only a few atypical cells. Abdominopelvic CT/MRI did not show enlargement of bilateral adnexa, endometrial thickenings lymphadenopathy, or ascites. Blood tests including tumor markers, CA125, CA19-9, and CEA were all within normal limits.

Because of the cytology/biopsy findings, hysterectomy and bilateral salphingo-oophorectomy with sampling of intra - pelvic lymph nodes were performed. During surgery, there were no visible lesions in the abdominopelvic space with only a small amount of yellowish to slightly muddy ascites. However, intraoperative cytology of the ascites demonstrated many adenocarcinoma nests (Fig. 1B).

**Figure 2:**
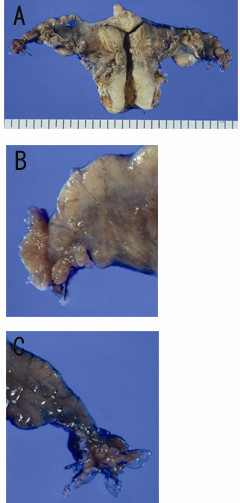
**Macroscopic findings of the surgical specimen**. (A) Front view of uterus and bilateral adnexa shows no remarkable findings except a few myoma in the uterine corpus. (B) Back view of left fimbrial ende shows shorter/denser fimbria compared to the right one. (C) Back view of the right fimbrial ende.

The surgical specimen showed bilateral, normal - appearing ovary and uterus with smooth/thin endometrium and a few myoma (Fig. 2A). There was no sign of disseminated tumor. Interestingly, compared to the right fimbrial ende, the left one was composed of shorter/dense fimbria when observed from the back-side (Fig. 2B, C).

Since there was no evident adenocarcinomatous lesion was on gross inspection, the whole surgical specimen was cut into histological section, in order to find the origin of the tumor. Bilateral adnexae except fimbria were cut into section at 5 mm intervals and each fimbriated end was cut sagitally into two.

**Figure 3:**
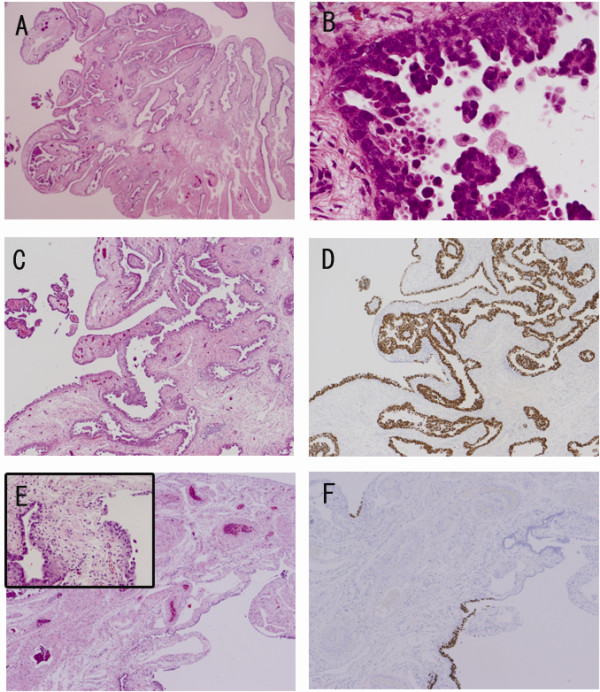
**Histological findings of the left fimbriated ende**. (A) Low - power view shows lining of enlarged cells at fimbrial surface with slightly thickened stroma. (B) High - power view shows serous adenocarcinoma in micropapillary structure. (C and D) Tumor cells shows immunopositive for P53. Fig. 3C and 3D are from the same site. (E and F) So-called p53 signature is seen at the periphery of the left fimbrial ende. Fig. 3E and 3F are from the same site. As seen in the Inset of E, p53-positive cells are composed of seemingly, non-cancerous cells. [A; H&E, ×4, B; H&E, ×40, C: H&E, ×15, D; Immunohistochemistry for p53, ×15, E: H&E, ×10, F: Immunohistochemistry for p53, ×10].

Pathologically, intraepithelial, non-invasive adenocarcinoma was found solely in the left fimbria (Fig. 3A-C). There was no tumor in the uterine cavity, right adnexa, lymph nodes, or left adnexa other than the fimbriated end. Immunohistochemically, adenocarcinoma was positive for p53 (Fig. 3D) and negative for calretinin/mesothelin. At the periphery of the left fimbrial ende, more than 12 normal - appearing nonciliated epithelial cells were positive for p53 in a consecutive fashion, so-called "p53 signatures" [7] (Fig. 3E, F).

## Discussion

Assignment of the primary site for PSCs is often problematic, since these lesions are usually discovered after spreading to the peritoneal surfaces. Our case was diagnosed to be tubal, especially, of fimbrial-origin. Surprisingly, despite the 'non-invasive' stage, our case showed spread to the peritoneal/intrauterine cavity, suggesting the ability of this type of tumor to spread rapidly. Although the peritoneum was not at all resected during surgery, the peritoneal surface is unlikely to have been primary site in our case for the following reasons. (1) There was no grossly suspicious lesion observed during the surgery. (2) Diffuse replacement of the fimbrial epithelium by the tumor and existence of a p53 signature may support the fimbria as the primary site. Only the co-exsistence of p53 signature may not be a strong evidence for its primary site without genetic testing, the diffuse replacement of the tumor suggested 'primary' rather than 'implantation'.

Until recently, most pathologists have not customarily examined the entire fallopian tube in cases of pelvic serous carcinoma, but rather only a perfunctory section of the central fallopian tube. Lee et al. [8] developed a protocol for sectioning and extensively examining the fimbriated end (SEE-FIM) to examine adnexal lesions thoroughly. They proposed application of this protocol to BRCA+ patients, patients with a family history of ovarian/breast cancer, and epithelial malignancies of the reproductive tract. We again recommend examining the fimbriae in PSC cases. As for the examination of routine hysterectomy cases, we think, it may be reasonable to examine bilateral fimbriae and 1 or 2 sections of bilateral tube.

As far as we know, there has no case report of serous adenocarcinoma of the fimbria at non-invasive (intraepithelial) stage, except prophylactically operated cases. In this case, the endometrial cytology contributed in the earlier detection of the fimbrial carcinoma.

In our case, there was a marked gross difference between the left fimbria (with adenocarcinoma) and the right fimbria (without), when seen observed from the back side. Detailed gross examination may be helpful for the early detection of fimbrial carcinoma. In our routine work, because of the surgical procedure, fimbriae are sometimes very much congested when submitting to pathological department. Earlier observation of fimbriae may be recommendable for their detailed gross examination.

## Conclusion

Clinical case detecting fimbrial adenocarcinoma at intraepithelial stage is very rare. This case report may suggest the benefit of endometrial cytology, detailed examination of fimbria, and may provide evidence suggesting fimbrial intraepithelial adenocarcinoma is one cause of PSC.

## Consent

Written informed consent was obtained from the patient for publication of this case report and accompanying images.

## Competing interests

The authors declare that they have no competing interests.

## Authors' contributions

FY participated in conception of the idea, histological/gross evaluation, and drafted the manuscript. MA participated in histological/gross evaluation, NT and KM participated in clinical data collection. YT participated in conception of the idea and histological evaluation. All authors read and approved the final manuscript.
